# Analysis of Self-Regulation of Eating Behaviors within Polish Adolescents’ COVID-19 Experience (PLACE-19) Study

**DOI:** 10.3390/nu14081679

**Published:** 2022-04-18

**Authors:** Dominika Guzek, Dominika Skolmowska, Dominika Głąbska

**Affiliations:** 1 Department of Food Market and Consumer Research, Institute of Human Nutrition Sciences, Warsaw University of Life Sciences (SGGW-WULS), 159C Nowoursynowska Street, 02-776 Warsaw, Poland; 2 Department of Dietetics, Institute of Human Nutrition Sciences, Warsaw University of Life Sciences (SGGW-WULS), 159C Nowoursynowska Street, 02-776 Warsaw, Poland; dominika_skolmowska@sggw.edu.pl (D.S.); dominika_glabska@sggw.edu.pl (D.G.)

**Keywords:** eating behavior, self-regulation, self-regulation of eating behaviour questionnaire (SREBQ), population-based study, national study, adolescents, PLACE-19 Study

## Abstract

The self-regulation of eating behaviors (suppression of behavioral short-term impulse to consume food products in the interest of pursuing long-term weight goals), has been an important determinant for adopting a healthy lifestyle in the period of the COVID-19 pandemic in adults, but there have been no such studies conducted in the population of adolescents in this period. The aim of the presented study is to analyze self-regulation of eating behaviors in the population-based sample of Polish adolescents within the Polish Adolescents’ COVID-19 Experience (PLACE-19) Study. The Self-Regulation of Eating Behavior Questionnaire (SREBQ) was used to assess self-regulation of eating behaviors in the studied population of 1126 Polish adolescents (818 females and 308 males), aged 15–20, recruited based on a random quota sampling within a national sample. Based on the screening questions of the SREBQ, 145 individuals were excluded from the analysis. The participants of the study were categorized based on their gender, body mass index, body mass index change during the COVID-19 pandemic, and food products declared as tempting. The major factors associated with SREBQ score were body mass index change and tempting food products. The respondents losing weight during the COVID-19 pandemic were characterized by a higher SREBQ score than those maintaining stable body mass or gaining weight (3.4 vs. 3.2 vs. 3.2; *p* = 0.0001). The respondents declaring both sweet and salty food products as tempting were characterized by a lower SREBQ score than those declaring only sweet, only salty, or declaring no tempting products (3.2 vs. 3.4 vs. 3.4 vs. 3.4; *p* < 0.0001). The major factors associated with SREBQ categories were gender, body mass index change, and tempting food products. A higher share of respondents characterized by a high self-regulation of eating behaviors was observed for males than for females (27.4% vs. 18.8%; *p* = 0.0142); for respondents losing weight during the COVID-19 pandemic than for respondents gaining weight (25.9% vs. 15.5%; *p* = 0.0423); as well as for respondents declaring no tempting products than those declaring both sweet and salty food products (38.3% vs. 18.2%; *p* < 0.0001). It was concluded that the self-regulation of eating behaviors in adolescents is closely associated with food products perceived as tempting by them, as well as with gender. During the COVID-19 pandemic, the low self-regulation of eating behaviors was a significant determinant of the body mass gain. Taking this into account, female adolescents characterized by a low self-regulation of eating behaviors especially should be subjected to a dedicated intervention program to prevent overweight and obesity.

## 1. Introduction

Self-regulation of eating behaviors, also called self-control of eating behaviors, is defined as suppression of the behavioral short-term impulse to consume food products in the interest of pursuing long-term weight goals [1]. The general theoretical framework of the self-regulation of eating behaviors is associated with four components, including: desire (the strength of the urges being experienced by individual), conflict (the degree to which the individual perceives the desire as being opposite to their long term-goals), resistance (the degree to which the individual is able to override the desire), and enactment (the behavioral outcome of the motivational processes) [2].

A higher level of self-regulation of eating behaviors leads to a healthier diet being followed, and as a result, it may prevent excessive weight gain [3]. In general, the level of self-regulation of eating behaviors is lower in the case of obese individuals than in normal-weight ones [4,5]. At the same time, for excessive-body-mass individuals, their low self-regulation of eating behaviors may limit their possibility to lose weight, as even if they try to follow any diet, they may be more likely to overeat while compared with individuals with a higher self-regulation of eating behaviors [6].

The level of self-regulation of eating behaviors results from numerous determinants, including cognitive restraint, moderation, mindfulness, disinhibition, delayed gratification, emotions and moods, self-efficacy, social support, environment, and physical activity [7]. The development of self-regulation of eating behaviors in children is associated with their general self-regulatory competence, and is related to executive functions [8], while it results from genetic and environmental factors, as well as their interactions [9]. It is also modulated by interactions with parents, especially in the case of using food to regulate their children’s behavior [10].

For adolescents, development of self-regulation of eating behaviors is especially important to be prepared to withstand the obesogenic food environment [11]. Taking this into account, habitual regulation of eating behaviors is becoming an element of a proper appetite control within an early prevention of overweight and obesity [12], while the development of self-regulation of eating behaviors may be supported by improving one’s body image, but without perceiving it as an essential element of personal life [13].

The outbreak of Coronavirus Disease 2019 (COVID-19), as well as related social isolations and lockdowns, caused significant changes in eating habits in a population of adolescents [14]. At the same time, the studies conducted in a population of adults indicated that during the COVID-19 pandemic, a higher level of self-regulation of eating behaviors was an important determinant for adopting a healthy lifestyle in this period [15]. Moreover, it is also indicated that a higher level of stress may reduce the level of self-regulation of eating behaviors [16], while the COVID-19 pandemic is well-known to be associated with increased levels of psychological distress [17].

However, there have been no studies of self-regulation of eating behaviors in the population of adolescents during the COVID-19 pandemic conducted so far. Taking this into account, the aim of the presented study is to analyze self-regulation of eating behaviors in the population-based sample of Polish adolescents within the Polish Adolescents’ COVID-19 Experience (PLACE-19) Study.

## 2. Materials and Methods

### 2.1. Ethical Approval and Study Design

The presented study was conducted on the basis of the approval of the Ethics Committee of the Central Clinical Hospital of the Ministry of Interior and Administration in Warsaw (No. 2/2021). It was conducted in agreement with the guidelines of the Declaration of Helsinki. Each participant of the study and their parents/legal guardians provided their informed consent for participation.

The presented analysis was conducted by the Institute of Human Nutrition Sciences, Warsaw University of Life Sciences (WULS-SGGW) within the PLACE-19 Study. The PLACE-19 study was conducted in the population-based sample of Polish adolescents, recruited from the Polish secondary schools, aged 15–20 (being a typical age for secondary school students in Poland). The PLACE-19 Study assessed various behaviors during the COVID-19 pandemic: within the first phase of the study, hygienic and personal protective behaviors [18,19,20]; within second phase of the study, nutritional behaviors [21,22,23,24,25,26]; and within third phase, emotional behaviors [27].

### 2.2. PLACE-19 Study Population

The presented phase of the PLACE-19 Study was conducted from 21 January 2021 to 17 February 2021, as presented in the previous study [27].

The random quota sampling of secondary schools was conducted within voivodeships and counties—from each voivodeship (16 voivodeships in Poland) 5 random counties were sampled (total number of 80 counties sampled), and from each county 5 random secondary schools were sampled (total number of 400 secondary schools sampled). The principal of each sampled secondary school was informed about inviting the school to participate in the study, and if they agreed, the study was conducted; the principal invited students, received access to an electronic version of the questionnaire, and provided it to each participant of the study.

The following inclusion criteria were formulated:

– Students of the sampled secondary schools;
– Aged 15–20 years;
– Informed consent to participate in the study provided by students and their parents/legal guardians.

The following exclusion criteria were formulated:

– Participation in the previous phases of the PLACE-19 Study;
– Missing/unreliable data provided in the completed form.

The total number of 1126 secondary school students (818 females and 308 males) participated in the study, while they represented 19 secondary schools in all the regions of Poland.

Within the studied population, on the basis of the screening questions of the Self-Regulation of Eating Behaviour Questionnaire (SREBQ), applied within the study, 145 respondents were excluded from the further analysis of self-regulation of eating behaviors (100 females, 45 males), resulting in the final sample of 981 secondary school students (718 females and 263 males).

### 2.3. Applied Questionnaire

When the PLACE-19 Study was conducted, education in all secondary schools was suspended and remote learning was introduced, as per the decision of the Polish Ministry of Education [28]. Therefore, all the data were collected using the computer-assisted web interview (CAWI) method.

The self-regulation of eating behaviors was assessed using the Self-Regulation of Eating Behaviour Questionnaire (SREBQ), developed and validated by Kliemann et al. [29] for the population of United Kingdom, and also used in the population of Germany [30]. Due to the fact that no Polish version of SREBQ was previously developed, before the study the SREBQ was translated into Polish on the basis of the recommendations by the World Health Organization (WHO) [31]. The forward translation into Polish and backward translation into English was conducted and was followed by the expert panel assessment. The forward translation was conducted by a native Polish-speaking researcher who was familiar with the discipline and with the concept of self-regulation of eating behaviors, in order to maximize the attainment of semantic, idiomatic, cultural, and conceptual equivalence. The backward translation was conducted by an independent researcher without knowledge about aim of the study and about objective of the questionnaire. The expert panel assessment included a native Polish-speaking and fluent English-speaking researchers, and it was conducted to develop and polish the final Polish version of the questionnaire, on the basis of the forward and backward translation previously conducted.

The SREBQ is based on 5 questions about self-regulation of eating behaviors–formulated as statements describing specific behaviors declaring how often those behaviors are presented by the respondent. The respondent is asked to specify the frequency of presenting specific behaviors as never, rarely, sometimes, often, or always, which are attributed to 5-point Likert scale from 1 (never) to 5 (always). In the case of 3 reverse statements, the reverse scale is applied from 1 (always), to 5 (never). The following self-regulation of eating-behavior statements are formulated within SREBQ:

(1) I give up too easily on my eating intentions (reverse statement);
(2) I’m good at resisting tempting food;
(3) I easily get distracted from my eating intentions (reverse statement);
(4) If I am not eating in the way I intend to I make changes;
(5) I find it hard to remember what I have eaten throughout the day (reverse statement).

On the basis of the score for each statement, the total score is calculated as the mean score of the 5 statements. Afterwards, the total score may be interpreted while using the following cut-offs: <2.8—low self-regulation of eating behaviors; 2.8–3.6—medium self-regulation of eating behaviors; >3.6—high self-regulation of eating behaviors [29].

The additional element of the SREBQ are screening questions about food products that respondents find tempting, about intentions to not eat too many tempting food products and about intentions to have a healthy diet. Those questions are included in order to assess self-regulation of eating behaviors only for those respondents who either have the intention to have a healthy diet or have the intention to not eat many foods they find tempting [29].

On the basis of the multiple-choice question about tempting food products with the following suggested answers: chocolate, crisps, cakes, ice cream, bread/toast, fizzy drinks, biscuits, sweets, popcorn, pastries, pizza, fried foods, chips, other foods (to be specified), respondents were categorized as those indicating sweet products as tempting, those indicating salty products as tempting, and those indicating both sweet and salty products as tempting. The last possibility was to not indicate any product as tempting (to declare that they do not find any food product tempting) and those respondents formulated the additional subgroup.

The additional questions included into the applied questionnaire assessed self-reported body mass and body mass changes during the COVID-19 pandemic. Each respondent was asked about their current body mass and height (February 2021), as well as about their body mass and height before the COVID-19 pandemic. In order to allow secondary-school students easier recognition of the period before the COVID-19 pandemic, respondents were asked about a specific period defined as the time before transition into a remote-education system (March 2020).

On the basis of the provided information about current body mass and height, as well as about body mass and height before the COVID-19 pandemic, for each period the body mass was assessed based on the body mass index (BMI) calculation [32]. Afterwards, the respondents aged >18 years were categorized as underweight (BMI < 18.5 kg/m^2^), normal weight (18.5–24.9 kg/m^2^), overweight (25.0–30.0 kg/m^2^), or obese (>30.0 kg/m^2^), on the basis of BMI cut-offs by the WHO [32]. The respondents aged <18 years were categorized using Polish gender-specific and age-specific growth reference values [33] and using dedicated OLAF software by the Children’s Memorial Health Institute (Warsaw, Poland) [34]. On the basis of the obtained BMI percentile, respondents aged <18 years were categorized as underweight (BMI < 5th percentile), normal weight (5th–85th percentile), overweight (85th–95th percentile), or obese (>95th percentile) on the basis of BMI percentile cut-offs by the WHO [35].

The body mass changes during the COVID-19 pandemic were defined based on changes in BMI value (for respondents aged >18 years), or changes in BMI percentile (for respondents aged <18 years), and respondents were categorized as losing weight, maintaining stable weight, or gaining weight during the COVID-19 pandemic (from March 2020 to February 2021).

### 2.4. Statistical Analysis

In order to assess the internal reliability of the Polish version of the SREBQ, the standardized factor loadings within the confirmatory factor analysis (CFA) were analyzed, similarly as in the German study by Schmalbach et al. [30]. The model fit indices were obtained as follows: χ^2^, comparative fit index (CFI), Tucker–Lewis index (TLI), root-mean-square error of approximation (RMSEA), and standardized root-mean-square residual (SRMR). The cut-offs were applied as follows: *p* for χ^2^ > 0.05 [36]; CFI ≥ 0.90 [37]; TLI ≥ 0.95 [38]; RMSEA ≤ 0.06 [39]; SRMR ≤ 0.08 [37]. The model fit indices were as follows: χ^2^ = 6.658 (*p* = 0.155), CFI = 0.994, TLI = 0.985, RMSEA = 0.026, SRMR = 0.025, indicating positive validation of the model for the Polish version of the SREBQ.

The results obtained while using the SREBQ were compared in the subsamples of respondents stratified by the following characteristics:

– Gender: female (*n* = 718) and male respondents (*n* = 263),
– Body mass index: underweight (*n* = 32), normal weight (*n* = 729), overweight (*n* = 142), and obese respondents (*n* = 78),
– Body mass index change during the COVID-19 pandemic: losing weight (*n* = 270), maintaining stable weight (*n* = 460), and gaining weight respondents (*n* = 251),
– Tempting food products: respondents declaring sweet (*n* = 104), salty (*n* = 71), sweet and salty products (*n* = 725), as well as declaring no food products as tempting (*n* = 81).

The distribution was verified for its normality using the Shapiro–Wilk test. The results were analyzed using the Mann–Whitney U test, or the Kruskal–Wallis H test by ranks, followed by the Tukey post hoc HSD test with Bonferroni correction, as well as the chi^2^ test.

The statistical analysis was conducted using Statistica 13.3 (StatSoft Inc., Tulsa, OK, USA), with *p* ≤ 0.05 considered significant.

## 3. Results

The general characteristics of the sample studied within the PLACE-19 Study is presented in Table 1. It is observed that the age and body mass index of female and male respondents did not differ, but their body mass index change during the COVID-19 pandemic differed—a higher share of female respondents than male ones lost weight during the COVID-19 pandemic (*p* = 0.0082).

The self-regulation of eating behaviors score assessed using the SREBQ within the PLACE-19 Study and in subsamples is presented in Table 2. Within the studied sample, it was observed that the major factors associated with the self-regulation of eating behaviors score were body mass index change (*p* = 0.0003) and tempting food products (*p* < 0.0001), while neither gender nor body mass index were associated with it. The respondents losing weight during the COVID-19 pandemic were characterized by a higher self-regulation of eating behaviors than those maintaining stable body mass or gaining weight (SREBQ score of 3.4 vs. 3.2 vs. 3.2). At the same time, the respondents declaring both sweet and salty food products as tempting were characterized by a lower self-regulation of eating behaviors than those declaring only sweet, only salty, or declaring no tempting products (SREBQ score of 3.2 vs. 3.4 vs. 3.4 vs. 3.4).

The self-regulation of eating behaviors assessed using the SREBQ within the PLACE-19 Study and in subsamples stratified by gender is presented in Table 3. For the questions about giving up too easily on their eating intentions (*p* < 0.0001), being good at resisting tempting food (*p* = 0.0382), and easily getting distracted from the way they intend to eat, there were significant differences between female and male respondents (*p* < 0.0001). The female respondents declared giving up too easily on their eating intentions, not being good at resisting tempting food, and easily getting distracted from the way they intend to eat (all of them being associated with low self-regulation of eating behaviors) more often than male ones.

The self-regulation of eating behaviors assessed using the SREBQ within the PLACE-19 Study in subsamples stratified by body mass index is presented in Table 4. For the questions about giving up too easily on their eating intentions (*p* = 0.0046), easily getting distracted from the way they intend to eat (*p* = 0.0004), and finding it hard to remember what they have eaten throughout the day, there were significant differences dependent on body mass index (*p* = 0.0317). Both overweight and obese respondents declared giving up too easily on their eating intentions, easily getting distracted from the way they intend to eat, and finding it hard to remember what they have eaten throughout the day (all of them being associated with low self-regulation of eating behaviors) more often than underweight and normal-weight ones.

The self-regulation of eating behaviors assessed using the SREBQ within the PLACE-19 Study in subsamples stratified by body mass index change during the COVID-19 pandemic is presented in Table 5. For the questions about giving up too easily on their eating intentions (*p* < 0.0001), being good at resisting tempting food (*p* < 0.0001), easily getting distracted from the way they intend to eat (*p* < 0.0001), and making changes while they are not eating in the way they intend to, there were significant differences between respondents losing and gaining weight (*p* < 0.0001). The respondents gaining weight declared giving up too easily on their eating intentions, not being good at resisting tempting food, easily getting distracted from the way they intend to eat, and not making changes while they are not eating in the way they intend to (all of them being associated with low self-regulation of eating behaviors) more often than those losing weight or maintaining a stable weight.

The self-regulation of eating behaviors assessed using the SREBQ within the PLACE-19 Study in subsamples stratified by tempting food products is presented in Table 6. For the questions about giving up too easily on their eating intentions (*p* = 0.0209), being good at resisting tempting food (*p* < 0.0001), easily getting distracted from the way they intend to eat (*p* < 0.0001), making changes while they are not eating in the way they intend to (*p* = 0.0001), and finding it hard to remember what they have eaten throughout the day, there were significant differences between respondents declaring no tempting food products and declaring any tempting products (*p* = 0.0038). The respondents declaring any tempting products declared giving up too easily on their eating intentions, not being good at resisting tempting food, easily getting distracted from the way they intend to eat, not making changes while they are not eating in the way they intend to, and finding it hard to remember what they have eaten throughout the day (all of them being associated with low self-regulation of eating behaviors) more often than those declaring no tempting food products.

The self-regulation of eating-behavior categories assessed using the SREBQ within the PLACE-19 Study and in subsamples is presented in Table 7. Within the studied sample it was observed that the major factors associated with self-regulation of eating behavior categories were gender (*p* = 0.0142), body mass index change (*p* = 0.0423), and tempting food products (*p* < 0.0001), while body mass index was not associated with it. A higher share of respondents characterized by a high self-regulation of eating behaviors was observed for males than for females (27.4% vs. 18.8%); for respondents losing weight during the COVID-19 pandemic than for respondents gaining weight (25.9% vs. 15.5%); as well as for respondents declaring no tempting products than those declaring both sweet and salty food products (38.3% vs. 18.2%).

## 4. Discussion

Within the conducted study, the major factors associated with SREBQ score were body mass index change and tempting food products, while the major factors associated with SREBQ categories were gender, body mass index change, and tempting food products. The lower SREBQ level was observed more often in female respondents, respondents gaining weight during the COVID-19 pandemic, and respondents declaring both sweet and salty food products as tempting than in other subgroups.

Gender is an important determinant of food acceptance, food intake, perceived food healthiness, and food avoidance [40]. In adults, both gender and general self-regulation play an important role in influencing weight-related variables [41]. It is associated with the fact that the relation between age and food cravings is stronger in female than in male individuals [42]. Similarly, in adolescents aged 11–17 years, in girls a higher level of suppression and lack of cognitive appraisal influenced a higher intake of high-calorie food products, but such association was not observed in boys [7]. It corresponds with a higher frequency of declaring giving up too easily on their eating intentions, not being good at resisting tempting food, and easily getting distracted from the way they intend to eat observed in the conducted study for female than for male respondents. All the behaviors listed for female respondents are associated with low self-regulation of eating behaviors, which is confirmed by a higher share of respondents characterized by a high self-regulation of eating behaviors observed for males than for females. Interestingly, the higher share of respondents characterized by a high self-regulation of eating behaviors observed for males than for females (27.4% vs. 18.8%) was accompanied by no differences in mean SREBQ score. Taking this into account, only the analysis of the responses to individual questions of the SREBQ provided insight as to why these seemingly discrepant results occurred.

The association between self-regulation of eating behaviors and body mass index changes may have been expected, as a body mass gain (which may, but not necessarily, result in excessive body mass) is indicated as a potential consequence of the need for immediate pleasure of tempting food products accompanied by consumption driven by emotions, these being elements of a low self-regulation of eating behaviors [7]. In a number of studies of children and adolescents, an association between self-regulation of eating behaviors and body mass index changes was indicated. In the prospective cohort study by Francis and Susman [43] conducted in children aged 3–12 years, it was concluded that lower self-regulation in a 9-year period may contribute to excessive weight gain. The same conclusions were formulated in the longitudinal prospective study by Duckworth et al. [44], conducted in older group of adolescents aged 10–13 years, as a higher level of self-control protected participants of the study against weight gain in the specific period of transition from childhood to adolescence. Similar observations were formulated in the longitudinal prospective study by Seeyave et al. [45] in children aged 4–11 years, for the ability to delay gratification, as lower ability contributed to excessive weight gain in 7-year period.

Comparable associations between self-regulation of eating behaviors and body mass index changes are indicated for adults. In the study by Ouwehand and Papies [46] conducted in female participants, it was concluded that for normal body mass individuals, food temptations trigger successful self-regulation, while for excessive body mass individuals, food temptations cause them to give up their dietary goals. At the same time, in the study by Kliemann et al. [47] conducted in a group of obese patients from primary care practices in the United Kingdom, it was observed that a positive change in self-regulatory skills over 3 months of therapy was associated with a better effect of weight loss intervention. It corresponds with a higher frequency of declaring giving up too easily on their eating intentions, not being good at resisting tempting food, easily getting distracted from the way they intend to eat, and not making changes while they are not eating in the way they intend to. All the behaviors listed for respondents gaining weight during the COVID-19 pandemic are associated with low self-regulation of eating behaviors, which is confirmed by a higher share of respondents characterized by a high self-regulation of eating behaviors observed for participants losing weight during the COVID-19 pandemic than for those gaining weight, as well as higher SREBQ scores.

Interestingly, the associations were observed only for body mass index changes during the COVID-19 pandemic, but not for body mass index. It may be explained by the fact that body mass index change during the COVID-19 pandemic may be directly influenced by emotional eating in this period [27] or being stressed [17], and as a result may reduce self-regulation of eating behaviors [48]. At the same time, in general, body mass index is influenced by numerous determinants, including those existing before the COVID-19 pandemic, so finally, the influence of the self-regulation of eating behaviors may not be so strong.

Last but not least, self-regulation of eating behaviors was in the studied group associated with declaring tempting food products. This association is based on the well-known effect of the strength of temptation on self-control [49]. It results from the fact that temptation counteracts the general process of self-control associated with healthy eating and weight management [50]. In the case of adolescents, it is indicated that easy access to tempting food products may be associated with increased consumption, but this impact is moderated by self-regulation strategies to facilitate following a healthy diet [51]. Taking this into account, it may have been expected that if the temptation is stronger (in the studied group attributed to subgroups of respondents experiencing any temptations), their self-control may be reduced and the respondent may be not able to resist existing temptations. In the presented study, such difference was indicated in the comparison of respondents declaring no tempting products with those declaring both sweet and salty food products.

## 5. Conclusions

It was concluded that the self-regulation of eating behaviors in adolescents is closely associated with food products perceived by them as tempting, as well as with gender. During the COVID-19 pandemic, the low self-regulation of eating behaviors has been a significant determinant of the body mass gain. Taking this into account, female adolescents characterized by a low self-regulation of eating behaviors should especially be subjected to a dedicated intervention program to prevent overweight and obesity.

## Figures and Tables

**Table 1:** The general characteristics of the sample studied within the Polish Adolescents’ COVID-19 Experience (PLACE-19) Study for the self-regulation of eating behaviors (*n* = 981).

		Total *n* = 981	Female *n =* 718	Male *n* = 263	*p*
Age (years) ^1^	Mean ± SD	16.7 ± 1.2	16.7 ± 1.2	16.7 ± 1.1	0.7128
	Median (25th–75th)	17.0 * (16.0–18.0)	17.0 * (16.0–18.0)	16.5 * (16.0–17.0)	
Body mass index ^2^	Underweight	32 (3.3%)	23 (3.2%)	9 (3.4%)	0.2305
	Normal weight	729 (74.3%)	542 (75.5%)	187 (71.1%)	
	Overweight	142 (14.5%)	94 (13.1%)	48 (18.3%)	
	Obesity	78 (8.0%)	59 (8.2%)	19 (7.2%)	
Body mass index change ^2^	Losing weight	270 (27.5%)	215 (29.9%)	55 (20.9%)	0.0082
	Maintaining stable body mass	460 (46.9%)	318 (44.3%)	142 (54.0%)	
	Gaining weight	251 (25.6%)	185 (25.8%)	66 (25.1%)	
Tempting food products ^2^	Sweet products	104 (10.6%)	77 (10.7%)	27 (10.3%)	0.9280
	Salty products	71 (7.2%)	54 (7.5%)	17 (6.5%)	
	Sweet and salty products	725 (73.9%)	529 (73.7%)	196 (74.5%)	
	No tempting products	81 (8.3%)	58 (8.1%)	23 (8.7%)	

* Nonparametric distribution (verified using Shapiro–Wilk test, *p* ≤ 0.05); ^1^ verified using Mann–Whitney U test; ^2^ verified using chi^2^ test.

**Table 2:** The self-regulation of eating behaviors score assessed using the Self-Regulation of Eating Behaviour Questionnaire (SREBQ), within the Polish Adolescents’ COVID-19 Experience (PLACE-19) Study (*n* = 981) and in subsamples.

		Mean ± SD	Median (25th–75th)	*p*
Total; *n* = 981		3.3 ± 0.7	3.2 * (2.8–3.6)	-
Gender ^1^	Female; *n =* 718	3.3 ± 0.7	3.2 * (2.8–3.6)	0.3484
	Male; *n* = 263	3.2 ± 0.6	3.4 * (2.8–3.8)	
Body mass index ^2^	Underweight; *n* = 32	3.4 ± 0.7	3.4 * (3.0–3.6)	1.0000
	Normal; *n* = 729	3.2 ± 0.6	3.2 * (2.8–3.6)	
	Overweight; *n* = 142	3.2 ± 0.7	3.2 * (2.8–3.6)	
	Obesity; *n* = 78	3.2 ± 0.7	3.2 * (2.8–3.6)	
Body mass index change ^2^	Losing weight; *n* = 270	3.4 ± 0.7	3.4 * (3.0–3.8) ^a^	0.0003
	Maintaining stable body mass; *n* = 460	3.2 ± 0.7	3.2 * (2.8–3.6) ^b^	
	Gaining weight; *n* = 251	3.1 ± 0.6	3.2 * (2.8–3.6) ^b^	
Tempting food products ^2^	Sweet products; *n* = 104	3.4 ± 0.6	3.4 * (3.0–3.6) ^a^	<0.0001
	Salty products; *n* = 71	3.3 ± 0.7	3.4 * (3.0–3.8) ^ab^	
	Sweet and salty products; *n* = 725	3.2 ± 0.6	3.2 * (2.8–3.6) ^b^	
	No tempting products; *n* = 81	3.6 ± 0.7	3.4 * (3.2–4.0) ^a^	

* Nonparametric distribution (verified using Shapiro–Wilk test, *p* ≤ 0.05); ^1^ verified using Mann–Whitney U test; ^2^ verified using Kruskal–Wallis H test by ranks, followed by Tukey post hoc HSD test; ^a,b^ statistically significant differences between variables with different upper index letters.

**Table 3:** The self-regulation of eating behaviors assessed using the Self-Regulation of Eating Behaviour Questionnaire (SREBQ), within the Polish Adolescents’ COVID-19 Experience (PLACE-19) Study (*n* = 981) and in subsamples stratified by gender.

Answers Declared Based on Self-Regulation of Eating Behaviors Questionnaire (SREBQ)		Total *n* = 981	Female *n =* 718	Male *n* = 263	p *
I give up too easily on my eating intentions (reverse statement)	Always	77 (7.8%)	70 (9.7%)	7 (2.7%)	<0.0001
	Often	253 (25.8%)	177 (24.7%)	76 (28.9%)	
	Sometimes	306 (31.2%)	232 (32.3%)	74 (28.1%)	
	Rarely	191 (19.5%)	148 (20.6%)	43 (16.3%)	
	Never	154 (15.7%)	91 (12.7%)	63 (24.0%)	
I’m good at resisting tempting food	Never	82 (8.4%)	55 (7.7%)	27 (10.3%)	0.0382
	Rarely	241 (24.6%)	188 (26.2%)	53 (20.2%)	
	Sometimes	295 (30.1%)	224 (31.2%)	71 (27.0%)	
	Often	274 (27.9%)	194 (27.0%)	80 (30.4%)	
	Always	89 (9.1%)	57 (7.9%)	32 (12.2%)	
I easily get distracted from the way I intend to eat (reverse statement)	Always	62 (6.3%)	51 (7.1%)	11 (4.2%)	<0.0001
	Often	276 (28.1%)	206 (28.7%)	70 (26.6%)	
	Sometimes	268 (27.3%)	205 (28.6%)	63 (24.0%)	
	Rarely	182 (18.6%)	144 (20.1%)	38 (14.4%)	
	Never	193 (19.7%)	112 (15.6%)	81 (30.8%)	
If I am not eating in the way I intend to I make changes	Never	82 (8.4%)	54 (7.5%)	28 (10.6%)	0.0616
	Rarely	131 (13.4%)	86 (12.0%)	45 (17.1%)	
	Sometimes	264 (26.9%)	193 (26.9%)	71 (27%)	
	Often	291 (29.7%)	225 (31.3%)	66 (25.1%)	
	Always	213 (21.7%)	160 (22.3%)	53 (20.2%)	
I find it hard to remember what I have eaten throughout the day (reverse statement)	Always	65 (6.6%)	45 (6.3%)	20 (7.6%)	0.7661
	Often	228 (23.2%)	172 (24.0%)	56 (21.3%)	
	Sometimes	188 (19.2%)	135 (18.8%)	53 (20.2%)	
	Rarely	116 (11.8%)	88 (12.3%)	28 (10.6%)	
	Never	384 (39.1%)	278 (38.7%)	106 (40.3%)	

* Verified using chi^2^ test.

**Table 4:** The self-regulation of eating behaviors assessed using the Self-Regulation of Eating Behaviour Questionnaire (SREBQ), within the Polish Adolescents’ COVID-19 Experience (PLACE-19) Study (*n* = 981) in subsamples stratified by body mass index.

Answers Declared Based on Self-Regulation of Eating Behaviors Questionnaire (SREBQ)		Underweight *n* = 32	Normal Weight *n* = 729	Overweight *n* = 142	Obesity *n* = 78	p *
I give up too easily on my eating intentions (reverse statement)	Always	0 (0.0%)	53 (7.3%)	14 (9.9%)	10 (12.8%)	0.0046
	Often	12 (37.5%)	192 (26.3%)	36 (25.4%)	13 (16.7%)	
	Sometimes	8 (25%)	238 (32.6%)	39 (27.5%)	21 (26.9%)	
	Rarely	4 (12.5%)	128 (17.6%)	32 (22.5%)	27 (34.6%)	
	Never	8 (25.0%)	118 (16.2%)	21 (14.8%)	7 (9.0%)	
I’m good at resisting tempting food	Never	3 (9.4%)	57 (7.8%)	16 (11.3%)	6 (7.7%)	0.1063
	Rarely	2 (6.3%)	170 (23.3%)	41 (28.9%)	28 (35.9%)	
	Sometimes	12 (37.5%)	220 (30.2%)	43 (30.3%)	20 (25.6%)	
	Often	10 (31.3%)	215 (29.5%)	32 (22.5%)	17 (21.8%)	
	Always	5 (15.6%)	67 (9.2%)	10 (7.0%)	7 (9.0%)	
I easily get distracted from the way I intend to eat (reverse statement)	Always	2 (6.3%)	41 (5.6%)	11 (7.7%)	8 (10.3%)	0.0004
	Often	10 (31.3%)	213 (29.2%)	40 (28.2%)	13 (16.7%)	
	Sometimes	3 (9.4%)	209 (28.7%)	35 (24.6%)	21 (26.9%)	
	Rarely	4 (12.5%)	120 (16.5%)	31 (21.8%)	27 (34.6%)	
	Never	13 (40.6%)	146 (20.0%)	25 (17.6%)	9 (11.5%)	
If I am not eating in the way I intend to I make changes	Never	3 (9.4%)	64 (8.8%)	11 (7.7%)	4 (5.1%)	0.7840
	Rarely	4 (12.5%)	97 (13.3%)	18 (12.7%)	12 (15.4%)	
	Sometimes	10 (31.3%)	186 (25.5%)	39 (27.5%)	29 (37.2%)	
	Often	10 (31.3%)	223 (30.6%)	41 (28.9%)	17 (21.8%)	
	Always	5 (15.6%)	159 (21.8%)	33 (23.2%)	16 (20.5%)	
I find it hard to remember what I have eaten throughout the day (reverse statement)	Always	5 (15.6%)	44 (6.0%)	14 (9.9%)	2 (2.6%)	0.0317
	Often	6 (18.8%)	168 (23.0%)	36 (25.4%)	18 (23.1%)	
	Sometimes	4 (12.5%)	140 (19.2%)	22 (15.5%)	22 (28.2%)	
	Rarely	1 (3.1%)	88 (12.1%)	14 (9.9%)	13 (16.7%)	
	Never	16 (50.0%)	289 (39.6%)	56 (39.4%)	23 (29.5%)	

* Verified using chi^2^ test.

**Table 5:** The self-regulation of eating behaviors assessed using the Self-Regulation of Eating Behaviour Questionnaire (SREBQ), within the Polish Adolescents’ COVID-19 Experience (PLACE-19) Study (*n* = 981) in subsamples stratified by body mass index change during the COVID-19 pandemic.

Answers Declared Based on Self-Regulation of Eating Behaviors Questionnaire (SREBQ)		Losing Weight *n* = 270	Maintaining Stable Body Mass *n* = 460	Gaining Weight *n* = 251	p *
I give up too easily on my eating intentions (reverse statement)	Always	12 (4.4%)	32 (7.0%)	33 (13.1%)	<0.0001
	Often	88 (32.6%)	125 (27.2%)	40 (15.9%)	
	Sometimes	81 (30.0%)	151 (32.8%)	74 (29.5%)	
	Rarely	31 (11.5%)	85 (18.5%)	75 (29.9%)	
	Never	58 (21.5%)	67 (14.6%)	29 (11.6%)	
I’m good at resisting tempting food	Never	25 (9.3%)	36 (7.8%)	21 (8.4%)	<0.0001
	Rarely	43 (15.9%)	100 (21.7%)	98 (39%)	
	Sometimes	68 (25.2%)	157 (34.1%)	70 (27.9%)	
	Often	94 (34.8%)	127 (27.6%)	53 (21.1%)	
	Always	40 (14.8%)	40 (8.7%)	9 (3.6%)	
I easily get distracted from the way I intend to eat (reverse statement)	Always	11 (4.1%)	25 (5.4%)	26 (10.4%)	<0.0001
	Often	95 (35.2%)	134 (29.1%)	47 (18.7%)	
	Sometimes	68 (25.2%)	131 (28.5%)	69 (27.5%)	
	Rarely	31 (11.5%)	81 (17.6%)	70 (27.9%)	
	Never	65 (24.1%)	89 (19.3%)	39 (15.5%)	
If I am not eating in the way I intend to I make changes	Never	21 (7.8%)	43 (9.3%)	18 (7.2%)	<0.0001
	Rarely	24 (8.9%)	69 (15.0%)	38 (15.1%)	
	Sometimes	56 (20.7%)	120 (26.1%)	88 (35.1%)	
	Often	85 (31.5%)	138 (30.0%)	68 (27.1%)	
	Always	84 (31.1%)	90 (19.6%)	39 (15.5%)	
I find it hard to remember what I have eaten throughout the day (reverse statement)	Always	18 (6.7%)	31 (6.7%)	16 (6.4%)	0.4567
	Often	56 (20.7%)	106 (23.0%)	66 (26.3%)	
	Sometimes	44 (16.3%)	88 (19.1%)	56 (22.3%)	
	Rarely	33 (12.2%)	56 (12.2%)	27 (10.8%)	
	Never	119 (44.1%)	179 (38.9%)	86 (34.3%)	

* Verified using chi^2^ test.

**Table 6:** The self-regulation of eating behaviors assessed using the Self-Regulation of Eating Behaviour Questionnaire (SREBQ), within the Polish Adolescents’ COVID-19 Experience (PLACE-19) Study (*n* = 981) in subsamples stratified by tempting food products.

Answers Declared Based on Self-Regulation of Eating Behaviors Questionnaire (SREBQ)		Sweet Products *n* = 104	Salty Products *n* = 71	Sweet and Salty Products *n* = 725	No Tempting Products *n* = 81	p *
I give up too easily on my eating intentions (reverse statement)	Always	8 (7.7%)	3 (4.2%)	62 (8.6%)	4 (4.9%)	0.0209
	Often	26 (25.0%)	17 (23.9%)	185 (25.5%)	25 (30.9%)	
	Sometimes	37 (35.6%)	27 (38.0%)	223 (30.8%)	19 (23.5%)	
	Rarely	13 (12.5%)	12 (16.9%)	161 (22.2%)	5 (6.2%)	
	Never	20 (19.2%)	12 (16.9%)	94 (13.0%)	28 (34.6%)	
I’m good at resisting tempting food	Never	10 (9.6%)	9 (12.7%)	47 (6.5%)	16 (19.8%)	<0.0001
	Rarely	22 (21.2%)	10 (14.1%)	205 (28.3%)	4 (4.9%)	
	Sometimes	27 (26.0%)	24 (33.8%)	230 (31.7%)	14 (17.3%)	
	Often	33 (31.7%)	22 (31.0%)	194 (26.8%)	25 (30.9%)	
	Always	12 (11.5%)	6 (8.5%)	49 (6.8%)	22 (27.2%)	
I easily get distracted from the way I intend to eat (reverse statement)	Always	3 (2.9%)	3 (4.2%)	53 (7.3%)	3 (3.7%)	<0.0001
	Often	27 (26.0%)	23 (32.4%)	201 (27.7%)	25 (30.9%)	
	Sometimes	24 (23.1%)	23 (32.4%)	207 (28.6%)	14 (17.3%)	
	Rarely	21 (20.2%)	7 (9.9%)	147 (20.3%)	7 (8.6%)	
	Never	29 (27.9%)	15 (21.1%)	117 (16.1%)	32 (39.5%)	
If I am not eating in the way I intend to I make changes	Never	13 (12.5%)	7 (9.9%)	50 (6.9%)	12 (14.8%)	0.0001
	Rarely	10 (9.6%)	6 (8.5%)	108 (14.9%)	7 (8.6%)	
	Sometimes	24 (23.1%)	17 (23.9%)	208 (28.7%)	15 (18.5%)	
	Often	25 (24.0%)	20 (28.2%)	228 (31.4%)	18 (22.2%)	
	Always	32 (30.8%)	21 (29.6%)	131 (18.1%)	29 (35.8%)	
I find it hard to remember what I have eaten throughout the day (reverse statement)	Always	5 (4.8%)	2 (2.8%)	55 (7.6%)	3 (3.7%)	0.0038
	Often	17 (16.3%)	19 (26.8%)	180 (24.8%)	12 (14.8%)	
	Sometimes	18 (17.3%)	11 (15.5%)	147 (20.3%)	12 (14.8%)	
	Rarely	14 (13.5%)	6 (8.5%)	90 (12.4%)	6 (7.4%)	
	Never	50 (48.1%)	33 (46.5%)	253 (34.9%)	48 (59.3%)	

* Verified using chi^2^ test.

**Table 7:** The self-regulation of eating-behavior categories assessed using the Self-Regulation of Eating Behaviour Questionnaire (SREBQ), within the Polish Adolescents’ COVID-19 Experience (PLACE-19) Study (*n* = 981) and in subsamples.

	Low *n* = 161	Medium *n* = 613	High *n* = 207	p *
Total; *n* = 981	161 (16.4%)	613 (62.5%)	207 (21.1%)	-
Female; *n =* 718	121 (16.9%)	462 (64.3%)	135 (18.8%)	0.0142
Male; *n* = 263	40 (15.2%)	151 (57.4%)	72 (27.4%)	
Underweight; *n* = 32	6 (18.8%)	19 (59.4%)	7 (21.9%)	0.6916
Normal; *n* = 729	110 (15.1%)	464 (63.6%)	155 (21.3%)	
Overweight; *n* = 142	29 (20.4%)	85 (59.9%)	28 (19.7%)	
Obesity; *n* = 78	16 (20.5%)	45 (57.7%)	17 (21.8%)	
Losing weight; *n* = 270	36 (13.3%)	164 (60.7%)	70 (25.9%) ^a^	0.0423
Maintaining stable body mass; *n* = 460	81 (17.6%)	281 (61.1%)	98 (21.3%) ^ab^	
Gaining weight; *n* = 251	44 (17.5%)	168 (66.9%)	39 (15.5%) ^b^	
Sweet products; *n* = 104	10 (9.6%) ^a^	69 (66.3%)	25 (24.0%) ^ab^	0.0001
Salty products; *n* = 71	10 (14.1%) ^ab^	42 (59.2%)	19 (26.8%) ^ab^	
Sweet and salty products; *n* = 725	136 (18.8%) ^b^	457 (63.0%)	132 (18.2%) ^a^	
No tempting products; *n* = 81	5 (6.2%) ^a^	45 (55.6%)	31 (38.3%) ^b^	

* Verified using chi^2^ test; ^a,b,c^ statistically significant differences between variables with different upper index letters in columns.
